# A new family of four-ring bent-core nematic liquid crystals with highly polar transverse and end groups

**DOI:** 10.3762/bjoc.9.4

**Published:** 2013-01-07

**Authors:** Kalpana Upadhyaya, Venkatesh Gude, Golam Mohiuddin, Rao V S Nandiraju

**Affiliations:** 1 Chemistry Department, Assam University, Silchar-788011, Assam, India, phone 919435522541, fax 913842270806

**Keywords:** bent-core mesogens, cyanobiphenyl, dipole moment, liquid crystals, nematic phase, polarity

## Abstract

Non-symmetrically substituted four-ring achiral bent-core compounds with polar substituents, i.e.., chloro in the bent or transverse direction in the central core and cyano in the lateral direction at one terminal end of the molecule, are designed and synthesized. These molecules possess an alkoxy chain attached at only one end of the bent-core molecule. The molecular structure characterization is consistent with data from elemental and spectroscopic analysis. The materials thermal behaviour and phase characterization have been investigated by differential scanning calorimetry and polarizing microscopy. All the compounds exhibit a wide-ranging monotropic nematic phase.

## Introduction

Following the discovery of chiral and polar properties of mesomorphic bent-core compounds [1–6] the area of design, synthesis and properties of banana or bent-shaped liquid crystals (LC) has attracted considerable attention from different research groups in the past two decades. Bent-core compounds that exhibit mesomorphic properties were first reported by Vorlander [7–8] and later by Matsunaga et al. [9–11]. Later the mesophases were confirmed to be banana-type phases [12–13]. The important properties of these compounds, that is, the ferri- and antiferro-electric phases, chirality and non-linear properties were recently explored [14–18] due to their possible utility in display devices. The majority of these bent-core compounds consist of five-, six- or seven-ring systems and exhibit the so-called banana phases. However, there are relatively few examples reported in the literature based on bent-core compounds exhibiting a nematic phase [6,19–24] and in particular with possible ferroelectric switching [25–26] and a biaxial nematic phase [27–30]. The nematic phases exhibited by bent-core compounds are distinctly different from the nematic phases exhibited by rodlike (calamitic) molecules.

Recent reports of a nematic phase composed of SmC-type cybotactic clusters [20–25] particularly in bent-core compounds followed by the optical observation of a biaxial nematic phase [27] motivated the design and synthesis of new bent-core compounds with different functional moieties to exhibit nematic phases. However, there are relatively few examples reported in the literature based on four-ring bent-core or nonlinear molecular architectures [31–39]. Furthermore, the introduction of a polar cyanobiphenyl moiety in bent-core systems exhibiting mesomorphism is rare [22].

## Design of the molecule

The recent experimental support in favour of a biaxial nematic phase that is exhibited by bent-core mesogens composed of two rod-like mesogenic wings coupled to a central linking moiety has been debated very well. The central linking moiety is mainly thiadiazole or oxadiazole derivatives with a large transverse dipole and an obtuse bent angle between the two arms. This represents a banana or V-shaped molecule composed of two uniaxial arms with a central transverse dipole. The study of the influence of dipole–dipole correlations on the stability of the biaxial nematic phase, in the two-particle-cluster approximation [40–41], revealed that (a) the polar-molecular-shape correlations between neighbouring molecules substantially favour the stabilization of biaxial nematic phases, and (b) the electrostatic interactions between permanent transverse dipoles of bent-core molecules also significantly stabilize the biaxial nematic phases. The introduction of a 2-chloro group in the 1,3-disubstituted phenyl ring of a bent-core molecular architecture can generate an obtuse bond angle of ~145°, which gives rise to an increase in bend angle as well as a strong dipole in the bending direction. The reduced bend from 120 to ~145° of the 1,3-phenyl moiety by the introduction of a chloro group in the 2-position and with the decrease in the number of rings from five or more to four in the molecular unit, places these compounds at the borderline between classical rodlike LCs and bent-core mesogens. The four-ring molecules can also be recognised as true hockey-stick model molecules. Further replacement of an azobenzene or salicylideneimine or phenylbenzoate unit in one of the arms of the bent molecule by a polar cyanobiphenyl moiety lends stiffness to the molecule with a strong dipole moment in the lateral direction. The realization of such a molecular architecture leads to a reduction in rotational disorder as well as a strong dipole in the bent direction. If molecular interactions are strong enough then the molecular structure can promote polar biaxial nematic phases.

**Scheme 1:**
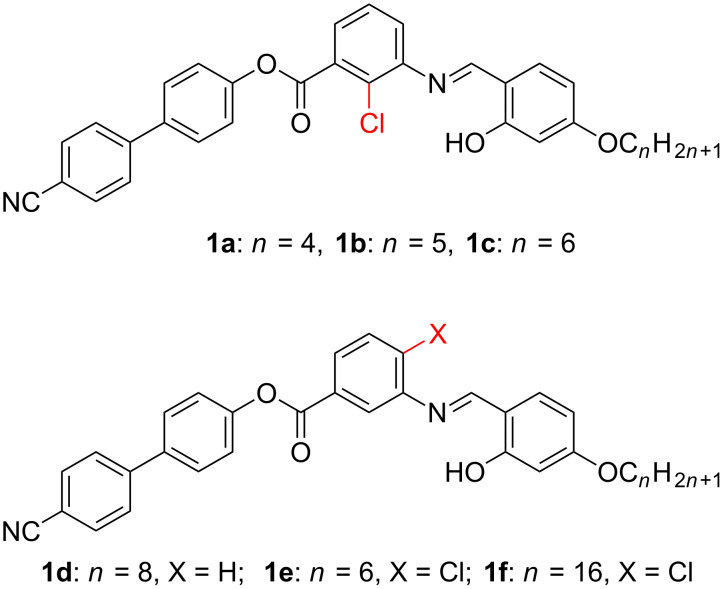
Molecular structures of the investigated four-ring bent-core compounds **1a–1f**.

In this study, we aimed to combine the lateral dipole in the form of a cyanobiphenyl moiety as one of the arms of the bent-core molecule, while the central bent core possesses a chloro substituent projected at a location inside the molecular core to represent the transverse dipole. The other end of the molecule is linked to 4-*n*-alkoxysalicylaldehyde through an imine moiety, which actually seems superior to the benzylidene aniline core and is more stable towards hydrolysis due to intramolecular hydrogen bonding. We report here the synthesis and characterization of the compounds **1a–1f** (Scheme 1) by elemental analysis, spectroscopic data, polarized optical microscopy (POM) and differential scanning calorimetry (DSC).

## Results and Discussion

### Synthesis and characterization

Here we adopted a very simple and straightforward synthetic methodology for the synthesis of these materials exhibiting a nematic phase. The non-symmetric four-ring molecules possess an alkoxy chain attached at only one end of the bent-core molecule, while the other arm ends with a highly polar cyano group. The end cyano group in one of the arms of **1a–1c** contributes to the large dipole moment. In highly polar calamitic cyanobiphenyl compounds [42–43] as well as in bent-core compounds possessing an end cyano moiety [44–48] the antiparallel short-range order was confirmed. The highly polarizable aromatic parts, which have conjugated electrons, contribute to the anisotropic dispersion potential between them. This is due to the arrangement of two neighbouring molecules in an antiparallel orientation and, hence, the aromatic moieties of the two molecules overlap. This contributes to the strong attractive interaction between them.

The molecule consists of two linkages between the three aromatic fragments, giving an ortho-hydroxy benzylidene moiety, a benzoic acid ester moiety, and a biphenyl residue. The salicylidene linkage instead of the unsubstituted benzylidene linkage was preferred due to the presence of the ortho-hydroxy group, which enhances the transverse dipole moment as well as the stability of the imines through intramolecular H-bonding to overcome the hydrolytic instability of the molecules towards moisture. The bent-core platform was designed from 3-substituted 2-chlorobenzoic acid and then coupled with 4'-hydroxy-[1,1'-biphenyl]-4-carbonitrile to achieve the target compound. The chloro substituent creates a strong transverse dipole moment, which may favour polar ordering.

**Scheme 2:**
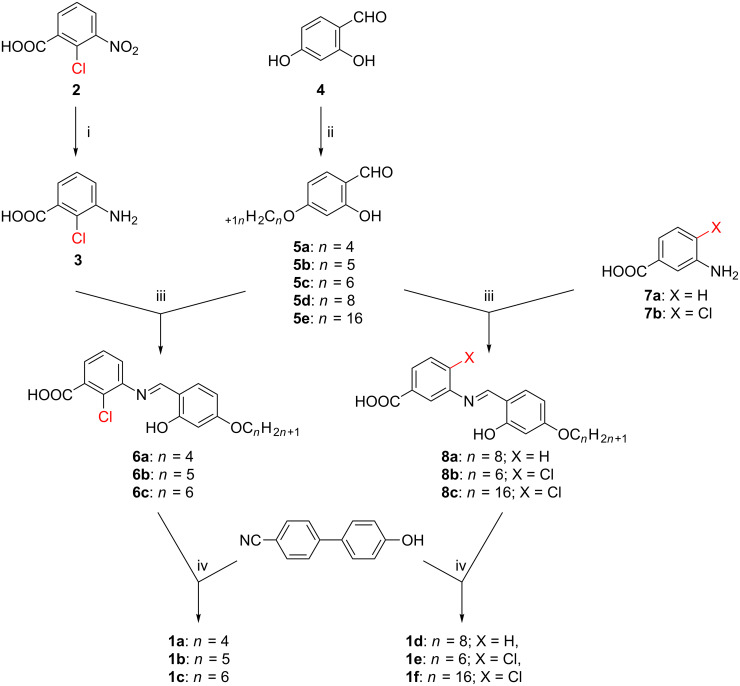
Synthetic details of the compounds **1a**–**1f**. Reagents and conditions: i. 5% Pd/C, H_2_, EtOAc stirring, 48 h; ii. dry acetone, KHCO_3_, C*_n_*H_2_*_n_*_+1_Br (*n* = 4, 5, 6, 8, 16); KI, Δ, 48 h; iii. abs EtOH, AcOH, Δ, 6 h; iv. DCC, DMAP, DCM, stirring, 48 h.

Commercially available 2-chloro-3-nitrobenzoic acid (**2**), is reduced with 5% Pd/C to yield 2-chloro-3-aminobenzoic acid (**3**). 4-*n*-Alkoxysalicylaldehydes **5a–5e** were synthesized following the procedures reported earlier [38–39]. 2-Chloro-3-aminobenzoic acid (**3**) was then condensed with alkoxysalicylaldehydes **5a**–5**c** to yield the corresponding 3-(4-*n*-alkoxysalicylidene)amino)-2-chlorobenzoic acids **6a**–**6c**. Coupling of these acids with 4’-hydroxy-[1,1'-biphenyl]-4-carbonitrile in the presence of *N*,*N*’-dicyclohexylcarbodiimide (DCC) and a catalytic amount of dimethylaminopyridine (DMAP) produced the desired products 4'-cyanobiphenyl-4-yl 2-chloro-3-[((2-hydroxy-4-*n*-alkoxybenzylidene)amino)benzoates **1a–1c**. To make a comparison with the mesomorphic **1a–1c**, other compounds **1d–1f** were prepared following the same procedure adopted for **1a** by using the appropriate reagents as outlined in Scheme 2.

The chemical structures of the final compounds **1** were confirmed by spectral techniques and elemental analysis. The analytical data are in good agreement with their chemical structures. The main observed FTIR peaks confirmed the intramolecular H-bonding of OH^…^N at 3184~3186 cm^−1^, CN stretching at 2220 cm^−1^, C=O stretching of an ester at 1737~1741 cm^−1^, C=N stretching of an imine at 1602~1606 cm^−1^, C=C stretching of an aromatic ring 1490 cm^−1^ and C–O–C stretching of an ester at ~1290 and ~1170 cm^−1^. The importance of the resorcylidene aniline core, present in calamitic ferroelectric liquid crystals [49–53], apparently seems superior to that of the benzylidene aniline core with respect to mesogenicity to exhibit an anticlinic bilayer smectic phase (SmAP_A_), and to its stability towards hydrolysis.

**Figure 1:**
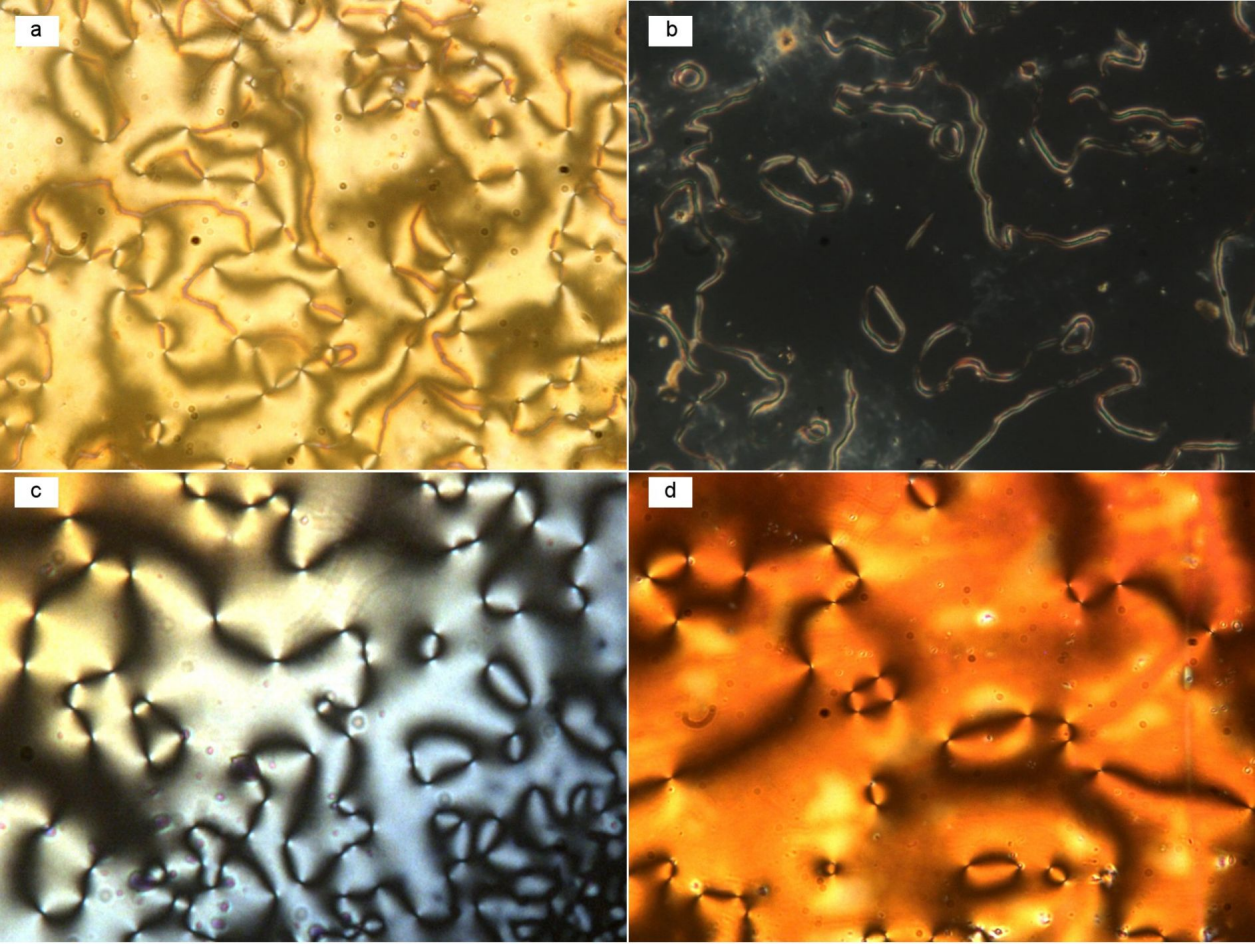
Microphotographs of compounds **1b** and **1c** in the nematic phase during the cooling cycle. (a) Birefringent Schlieren texture of **1b** at 145 °C; (b) disappearance of birefringence and transformation to the homeotropic texture of **1b** at 122 °C; (c) two-brush-defect Schlieren texture of **1c** at 161 °C from I–N transition; (d) Schlieren texture with increased birefringence at 156 °C.

The mesomorphic behaviour of compounds **1a**–**1c** was characterized by polarized optical microscopy (POM), and the samples, on cooling from the isotropic phase, exhibited marble or highly birefringent two-brush Schlieren textures (Figure 1) in the nematic phase. This indicates a predominantly homogeneous alignment of the sample with the nematic director being on average parallel to the substrate surface. On further cooling they exhibit a slow transition to a homeotropic alignment initially over small areas, which subsequently spread to the entire area under observation, as shown in Figure 1. The strong attraction between rigid cores augmented by intermolecular interactions due to end-polar moieties as well as the bent shape can promote the nearest neighbour aggregations and hence the formation of cybotactic clusters in the nematic phase [23,27]. Although the presence of cybotactic clusters in the nematic phase can clearly be demonstrated by small-angle X-ray studies due to their small size, such clusters cannot be observed by POM. Hence the dark regions of the optical texture (homeotropic texture, Figure 1b) can be explained by the growth of cybotactic clusters followed by the transformation to a homeotropic orientation [21]. The homeotropic regions exhibit transient birefringent textures either by shearing or tapping.

**Figure 2:**
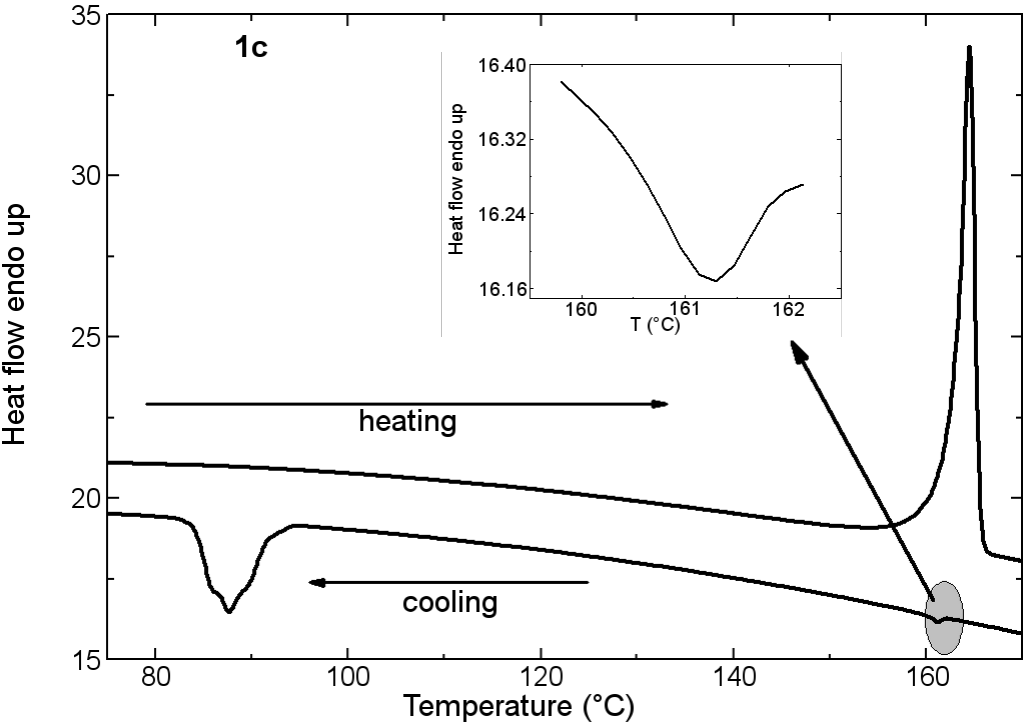
DSC trace of **1c** obtained during initial heating and cooling cycles scanned at a rate of 10 K/min.

The phase transitions are confirmed by differential scanning calorimetry (DSC). The thermodynamic data are presented in Table 1. A representative DSC trace obtained during the initial heating and cooling cycles scanned at a rate of 10 K min^−1^ for **1c** is presented in Figure 2. The nematic–isotropic phase-transition enthalpies are of the order of 0.15~0.29 kJ/mol for **1a–1c** and are in agreement with the reported enthalpies at the N–I phase transition exhibited by bent-core compounds.

**Table 1:** Phase-transition temperatures (°C) of the compounds **1a**–**1f**, recorded for second heating (first row) and second cooling (second row) cycles at 10 °C/min from DSC and confirmed by polarized optical microscopy. The enthalpies (∆*H* in kJ/mol) are presented in parentheses.

Compound	Phase transition temperatures (enthalpy)

**1a**	Cr 176.5 (56.1) Iso Cr 45.1 (2.05) N 143.3 (0.158) Iso
**1b**	Cr 176.8 (79.7) Iso Cr 78.8 (41.6) N 162.8 (0.227) Iso
**1c**	Cr 164.4 (58.8) Iso Cr 87.6 (63.5) N 161.3 (0.290) Iso
**1d**	Cr 144 Iso
**1e**	Cr 189 Iso
**1f**	Cr 148 Iso

To gain an understanding of the structure–property relationship we synthesized other homologues of the same core with variations in the position of the chloro substituent, or without any substituent. The unsubstituted homologue **1d** with a terminal *n*-octyloxy chain did not exhibit mesomorphism. Similarly, in cases where the chloro substituent was shifted to the 4-position, the compound **1e**, with a *n*-hexyloxy terminal chain, or its higher homologue with a *n*-hexadecyloxy chain **1f**, did not exhibit mesomorphism. It is surprising that the aspect ratio of all compounds is almost the same, but the compounds with a transverse chloro substituent exhibit monotropic nematic phases, while the other homologues with a lateral dipole did not exhibit mesomorphism. In general, the monotropic behaviour was often considered as kinetically unstable, and hence it could occur when the aspect ratio of the molecule was not appropriate. Further investigations are in progress to understand the reasons for such behaviour.

The size of the bent cores, reflected by the number of rings; lateral or transverse substituents, which promote lateral or transverse interactions; and the length of terminal aliphatic chains, which strongly influences the segregation of the aromatic and aliphatic regions, all contribute to the formation of banana mesomorphism, in particular the layered phases. The bent shape and size of the bent core with a lateral polar substituent [21–22,54–57] leads to the reduction of the rotational disorder of the molecules around their long axes to promote nematic phases. However, for short-chain members of a homologous series (*n*-butyl to *n*-hexyl units), the microsegregation of incompatible aromatic and aliphatic moieties of the molecules is weak, and hence nematic phases can be found rather than the layered phases. Furthermore, they promote nearest-neighbour aggregations, such as the cybotactic clusters in the nematic phase, which are also augmented by intermolecular interactions due to polar end moieties as well as the bent shape, reflecting the textures with homeotropic orientation. The chloro substituent in the transverse position of the bent molecule promoted mesomorphism. When the size of the bent core is reduced to four rings, the chloro substitution in the lateral position possibly contributes to a weakening of lateral interactions and hence does not promote the necessary molecular interactions to exhibit mesomorphism. The repulsion between the transverse polar chloro substituent in **1a–1c** and the adjacent ester or imine linkages leads to a torque exercised on the wing of the bent molecule and, hence, leads to an increase in the bending angle. An increase in bending angle may contribute to increased van der Waals interactions and dispersion forces and, hence, promote mesomorphism during the cooling cycle in these compounds. The absence of mesomorphism in **1e** and **1f** may be attributed to possibly weakened lateral interactions and increased intermolecular spacing between molecules.

### Density functional theory calculations

The quantum mechanical calculations of molecular properties in the gas phase were performed by using density functional theory (DFT) [58] employing the combination of the Becke (3-parameter)–Lee–Yang–Parr (B3LYP) hybrid functional and the 6-31g(d,p) basis set using the Gaussian 09 package, to obtain the information related to molecular conformation, bend angle, dipole moment, molecular polarizability, and asymmetry parameter of all the compounds **1a**–**1f**. Full geometry optimizations were carried out without imposing any constraints [58]. Spin-restricted DFT calculations were carried out in the framework of the generalized gradient approximation (GGA) by using the B3LYP hybrid functional, exchange-correlation functional and the 6-31G(d,p) standard basis set [59–60] due to its successful application to larger organic molecules, as well as hydrogen-bonded systems in the past [61–63] and bent-core molecules [30,64–67] recently.

**Figure 3:**
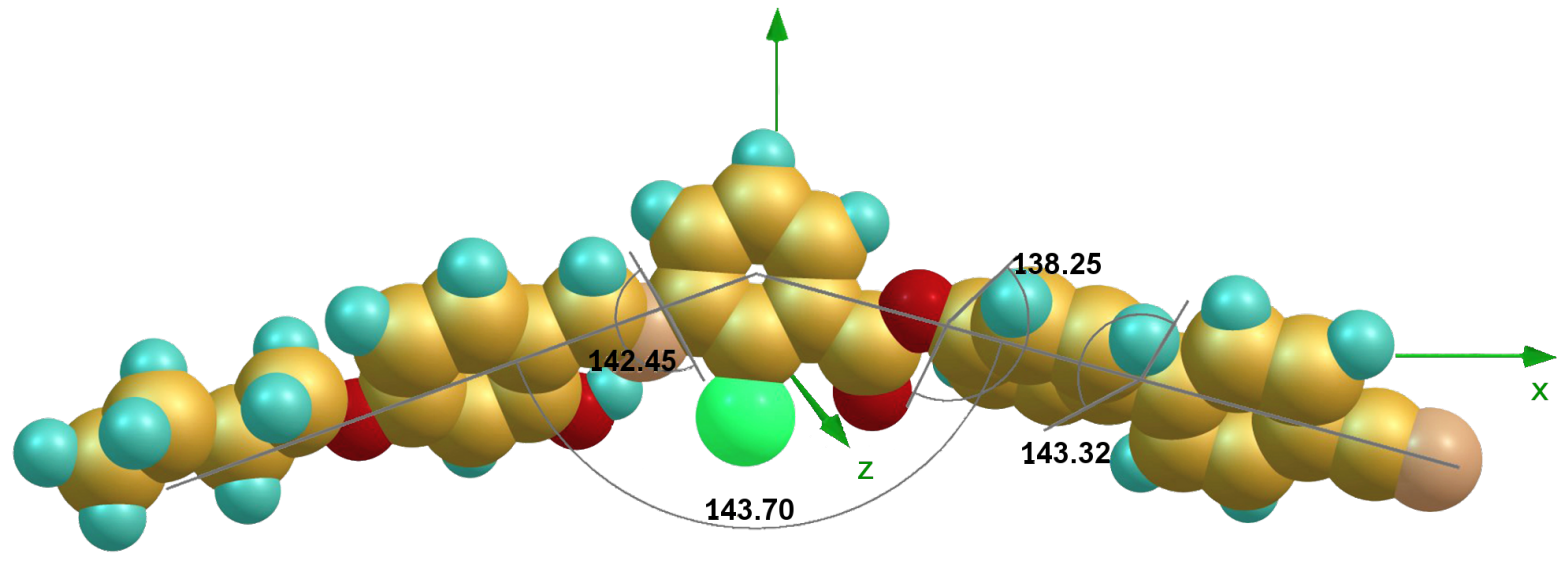
Molecular structure of **1a** optimized at the DFT level. The bent angle is 143°, and the three dihedral angles indicated the out-of plane adjacent phenyl rings.

The three dihedral angles between the first phenyl and central phenyl moieties with imine linkage, central ring and first biphenyl ring with ester linkage and between the two phenyl rings of biphenyl moiety are 142°, 138° and 143°, respectively (Figure 3), reflecting the absence of coplanarity of the phenyl rings in the molecule. The analysis shows that the bend angle is approximately 143° for all mesogens and is influenced by the transverse chloro substituent in the 2-position of the central phenyl ring. The bending angle for the compound **1d** is 138° and for **1e** and **1f** is 135°. This may have contributed to an increase in intermolecular separation and thereby did not promote mesomorphism. For all the mesogens, the dipole points almost in the lateral direction with respect to molecular long axis.

All molecules possess a considerably larger dipole along the molecule’s long axis (*x* axis). The results of bending angles and dipole-moment components are summarized in Table 2 and Table 3 for all the molecules. Furthermore, the polarizability component α_XX_ is largest along the longitudinal *x* axis (Table 3), which indicates a more dispersed electron cloud. Such a dispersed electron cloud leads to a larger amount of surface contact. The asymmetry parameter η = [(α_yy_ – α_zz_)/ (α_xx_ − α^iso^)] ~ 0.20 ± 0.02, (α_xx_, α_yy_, and α_zz_ being the principle component of the polarizability tensor and α^iso^ = (α_xx_ + α_yy_ + α_zz_)/3) is rather small reflecting the importance of the large bending angle. However, it decreases with the increase in end-chain length.

**Table 2:** DFT-calculated bend angle Θ, dipole-moment components (μ_x_, μ_y_, μ_z_), modulus (μ), and angle γ, formed with respect to the longitudinal axis *x* as shown in Figure 3.^a^

Compound	Dipole moment (Debye)				Bend angle (°)

	µ_x_	µ_y_	µ_z_	µ_resultant_^b^	

**1a**	8.60	5.97	2.47	10.76	143
**1b**	8.63	5.91	2.60	10.78	143
**1c**	8.59	5.98	2.77	10.83	143
**1d**	8.30	5.57	2.53	10.31	138
**1e**	7.14	3.39	3.01	8.46	135
**1f**	6.76	3.78	3.49	8.50	135

^a^The values relative to angles and dipole moment are expressed in degree (°) and Debye (D), respectively. ^b^µ_resultant_ = (µ_x_^2^ + µ_y_^2^ + µ_z_^2^)^1/2^.

**Table 3:** DFT calculated principal polarizability components (α_xx_, α_yy_, α_zz_), isotropic polarizability α^iso^ = (α_xx_ + α_yy_ + α_zz_)/3, polarizability anisotropy Δα = [α_xx_ − (α_yy_ + α_zz_)/2], and asymmetry parameter, η = [(α_yy_ – α_zz_)/ (α_xx_ − α^iso^)]. Parameters are relative to the molecular polarizability tensor in the Cartesian reference frame.^a^

Compound	α_xx_	α_yy_	α_zz_	α^iso^	∆α	η_α_

**1a**	780	301	222	435	518	0.228
**1b**	800	308	234	447	533	0.208
**1c**	817	315	245	459	537	0.195
**1d**	838	367	213	473	548	0.420
**1e**	783	360	218	454	494	0.431
**1f**	929	460	316	568	441	0.398

^a^All polarizability components and the anisotropy parameter are calculated in (bohr)^3^ (with 1 bohr = 0.52917 Å).

## Conclusion

Non-symmetric four-ring bent-core hockey-stick-shaped molecules have been designed and synthesized, and mesomorphism has been confirmed in these polar molecular architectures. The thermal behaviour of the novel four-ring compounds has been investigated by DSC and POM. The compounds derived from transverse and terminal polar substituents in the bent-core molecules exhibited monotropic nematic phases, while compounds with lateral and terminal polar substituents did not show mesomorphism. A preliminary insight into the structure–property relationships of bent-core molecules revealed that the mesophase behaviour is strongly influenced by the number of rings [21–22,35,54–57], the type and direction of linking groups [33–39], the nature and position of terminal and transverse substituents [39], and the type and length of the terminal chains [39]. As these results are of importance in relation to current commercial requirements for a range of stable bent-core mesogens exhibiting a wide spectrum of physical properties, further experimental studies, i.e., examining birefringence, elastic constants, viscosity, and electro-optical characteristics, are in progress in fundamental and applied research. Attempts to realize the enantiotropic mesomorphism in such compounds at ambient temperature by a modification of the substituents with different molecular topologies are currently in progress.

## Experimental

All the chemicals were procured from M/s Alfa Aesar or Aldrich or Tokyo Kasei Kogyo Co. Ltd. The solvents and reagents are of AR grade and were distilled and dried before use. Micro analysis of C and H elements were determined on Carlo-Erba 1106 elemental analyzer. IR spectra were recorded on Shimadzu IR Prestige-21, FTIR-8400S (ν_max_ in cm^−1^) as KBr disks. The ^1^H nuclear magnetic resonance spectra were recorded on NMR spectrometers (Bruker DPX-400 400 MHz or JEOL AL300 FTNMR 300 MHz) in CDCl_3_ solution (chemical shift δ in parts per million) with TMS as internal standard. The liquid-crystalline properties were observed and characterized by polarizing microscopy (Nikon optiphot-2-pol with an attached hot and cold stage HCS302, with STC200 temperature controller configured for HCS302 from INSTEC Inc. USA). The phase transition temperatures and associated enthalpies were recorded on differential scanning calorimetry (Perkin-Elmer Pyris-1 system) with a heating/cooling rate of 10 °C/min.

**4-*n*-Butyloxy-2-hydroxybenzaldehyde (5a):** In the synthesis of 4-*n*-butyloxy-2-hydroxybenzaldehyde (Scheme 2), monoalkylation was performed by using a modification of the literature procedure to improve the product yield. 2,4-Dihydroxybenzaldehyde (**4**, 10 g, 72.4 mmol), 1-bromobutane (10.3 mL, 75 mmol), KHCO_3_ (6.30 g, 75 mmol) and KI (catalytic amount) were mixed in dry acetone (250 mL) and then the mixture was heated under reflux for 48 h. It was then filtered hot to remove the insoluble solid. The warm solution was neutralized by the addition of dilute HCl, then extracted twice with CHCl_3_ (100 mL). The combined extracts were concentrated to give a purple solid. The product was purified by column chromatography using silica gel (60–120 mesh) eluting with a mixture of chloroform and hexane (V/V, 1/1) followed by evaporation of solvent. It gave the product as a pale yellow liquid. Yield = 10.6 g (70%); IR ν_max_: 1666 (ν_C=O_, aldehyde), 3449 (ν_O-H_, H-bonded) cm^−1^; ^1^H NMR (300 MHz ,CDCl_3_) δ 11.41 (s, 1H, -OH), 9.66 (s, 1H, -CH=O), 7.40 (d, 1H, *J* = 8.8 Hz, ArH), 6.51 (d, 1H, *J* = 8.9 Hz, ArH), 6.61 (d, 1H, *J* = 2.8 Hz, ArH), 4.03 (t, 2H, *J* = 7.8 Hz, -O-CH_2_-); 1.65 (q, 2H, *J* = 6.6 Hz, -OCH_2_-CH_2_-), 1.38–1.20 (m, 4H, -(CH_2_)_2_-), 0.88 (t, 3H, *J* = 6.6 Hz, -CH_3_).

The other compounds **5b–5e** had been synthesized following the procedure adopted for **5a**.

**2-Chloro 3-*N*-(4-*n*-butyloxysalicylidene)aminobenzoic acid (6a):** An ethanolic solution of 2-chloro-3-aminobenzoic acid (**3**, 0.51 g, 3 mmol) was added to an ethanolic solution (20 ml) of 4-*n*-butyloxysalicylaldehyde (**5a**, 0.58 g, 3 mmol). The mixture was heated under reflux with a few drops of glacial acetic acid as catalyst for 6 h to yield the yellow-coloured Schiff’s base. The precipitate was collected by filtration from the hot solution and recrystallized several times from absolute ethanol to give a pure compound. Yield = 0.8 g (84%); IR ν_max_: 1618 (ν_CH=N_, imine), 1719 (ν_C=O_, acid), 3425(ν_O-H_, H-bonded) cm^−1^; ^1^H NMR (400 MHz, CDCl_3_): δ 13.49 (s, 1H, -OH), 9.99 (s, 1H, -COOH), 8.35 (s, 1H, -CH=N-), 7.88 (d, *J* = 8.4 Hz, 1H, ArH), 7.33 (t, *J* = 8.0 Hz, 1H, ArH), 7.46 (d, *J* = 8.4 Hz, 1H, ArH), 7.29 (d, *J* = 7.8 Hz, 1H, ArH), 6.98 (d, *J* = 8.4 Hz, 1H, ArH), 6.43 (s, 1H, ArH), 4.01 (t, *J* = 7.8 Hz, 2H, -OCH_2_-), 1.56 (q, 2H, -CH_2_-), 1.29–1.21 (m, 2H, -CH_2_), 0.88 (t, 3H, *J* = 7.8 Hz, -CH_3_).

The compounds **6b**, **6c**, **8a–8c** were synthesized following the procedure adopted for **6a**, with appropriate starting materials.

**4'-Cyano-[1,1'-biphenyl]-4-yl 3-((4-butoxy-2-hydroxybenzylidene)amino)-2-chlorobenzoate (1a): ***N*,*N*'-dicyclohexylcarbodiimide (DCC, 0.206 g, 1.0 mmol) was added all at once to a stirred mixture of 3-((4-*n*-butyloxy-2-hydroxybenzylidene)amino)-2-chlorobenzoic acid (0.35 g, 1.0 mmol), 4'-hydroxy-[1,1'-biphenyl]-4-carbonitrile (0.195 g, 1.0 mmol) and a catalytic amount of *N*,*N*'-dimethylaminopyridine (DMAP) dissolved in dry dichloromethane (DCM) (50 mL) in a round-bottom two-neck flask flushed with N_2_, arranged with a teflon-coated magnetic stirrer at room temperature. The reaction mixture was stirred for 48 h at room temperature (completion of reaction was confirmed by TLC analysis) followed by the removal of dicyclohexylurea by filtration. Evaporation of the solvent in vacuum gave the crude product, which was washed with hot ethanol, followed by recrystallization from ethanol/ethyl acetate to obtain the pure product **1a** as a yellow solid. Yield = 0.30 g (58%); IR (KBr) ν_max_: 3186, 2951, 2864, 2221, 1741, 1604, 1512, 1490, 1384, 1344, 1288, 1220, 1192, 1163, 1107, 744 cm^−1^; ^1^H NMR (400 MHz, CDCl_3_) δ 13.46 (s, 1H, -OH), 8.55 (s, 1H, -CH=N-), 7.86 (dd, *J* = 2.0, 7.6 Hz, 1H, ArH), 7.75 (dd, *J* = 2.4 Hz, 8.8 Hz, 2H, ArH), 7.69 (dd, *J* = 2.0, 8.8 Hz, 2H, ArH), 7.64 (dd, *J* = 2.4 Hz, 8.8 Hz, 2H, ArH), 7.46 (d, *J* = 8.0 Hz, 1H, ArH), 7.42 (d, *J* = 8.0 Hz, 1H, ArH), 7.38 (dd, *J* = 2.4, 8.0 Hz, 1H, ArH), 7.31 (dd, *J* = 2.0, 7.6 Hz, 2H, ArH), 6.52 (dd, *J* = 2.4 Hz, 7.2 Hz, 2H, ArH), 4.02 (t, *J* = 6.4 Hz, 2H, -OCH_2_-), 1.83–1.76 (m, 2H, -(CH_2_)-), 1.55–1.46 (m, 2H, -(CH_2_)-), 0.98 (t, 3H, -CH_3_); Anal. calcd for C_31_H_25_ClN_2_O_4_: C, 70.92; H, 4.80; found: C, 70.61; H, 4.75.

For the synthesis of **1b–1f**, the same experimental procedure as described for the preparation of **1a (**with appropriate chemicals as detailed in Scheme 2) was followed to obtain the yellow solids. Yield of **1b** = 0.32 g, 60%, yield of **1c** = 0.30 g, 55% ; yield of **1d** = 0.31 g, 56%; yield of **1e** = 0.33 g, 61%; yield of **1f** = 0.45 g, 65%.

**4'-Cyano-[1,1'-biphenyl]-4-yl 3-((4-(pentyloxy)-2-hydroxybenzylidene)amino)-2-chlorobenzoate (1b):** IR (KBr) ν_max_: 3186, 2951, 2864, 2218, 1739, 1602, 1512, 1487, 1392, 1344, 1290, 1247, 1219, 1192, 1166, 1107, 748 cm^−1^; ^1^H NMR (400 MHz, CDCl_3_) δ 13.46 (s, 1H, -OH), 8.55 (s, 1H, -CH=N-), 7.86 (dd, *J* = 2.0, 7.2 Hz, 1H, ArH), 7.75 (d, *J* = 8.0 Hz, 2H, ArH), 7.70 (d, *J* = 8.4 Hz, 2H, ArH), 7.67 (d, *J* = 8.8 Hz, 2H, ArH), 7.47 (d, *J* = 8.0 Hz, 1H, ArH), 7.45 (dd, *J* = 2.8, 7.6 Hz, 1H, ArH), 7.42 (d, *J* = 8.0 Hz, 1H, ArH), 7.32 (d, *J* = 8.8 Hz, 2H, ArH), 6.53 (dd, *J* = 2.4, 8.8 Hz, 2H, ArH), 4.01 (t, 2H, *J* = 6.4 Hz, -OCH_2_-), 1.84–1.77 (q, 2H, -(CH_2_)-), 1.48–1.34 (m, 4H, -(CH_2_)_2_-), 0.99 (t, *J* = 6.8 Hz, 3H, -CH_3_); Anal. calcd for: C_32_H_27_ClN_2_O_4_: C, 71.30; H, 5.05; found: C, 71.18; H, 4.99.

**4'-Cyano-[1,1'-biphenyl]-4-yl 3-((4-(hexyloxy)-2-hydroxybenzylidene)amino)-2-chlorobenzoate (1c):** IR (KBr) ν_max_: 3184, 2951, 2856, 2222, 1737, 1600, 1512, 1487, 1386, 1342, 1290, 1215, 1192, 1170, 1112, 746 cm^−1^; ^1^H NMR (400 MHz, CDCl_3_) δ 13.43 (s, 1H, -OH), 8.54 (s, 1H, -CH=N-), 7.85 (dd, *J* = 2.0, 7.2 Hz, 1H, ArH), 7.74 (d, *J* = 8.4 Hz, 2H, ArH), 7.69 (d, *J* = 8.8 Hz, 2H, ArH), 7.66 (d, *J* = 8.4 Hz, 2H, ArH), 7.45 (d, *J* = 8.0 Hz, 1H, ArH), 7.33 (dd, *J* = 2.8, 8.0 Hz, 1H, ArH), 7.39 (d, *J* = 8.4 Hz, 1H, ArH), 7.31 (d, *J* = 8.8 Hz, 2H, ArH), 6.53 (dd, *J* = 2.0, 8.8 Hz, 2H, ArH), 4.02 (t, 2H, *J* = 6.4 Hz, -OCH_2_-), 1.85–1.78 (q, 2H, -(CH_2_)-), 1.49–1.35 (m, 6H, -(CH_2_)_3_-), 0.92 (t, *J* = 6.8 Hz, 3H, -CH_3_); Anal. calcd for C_33_H_29_ClN_2_O_4_: C, 71.67; H, 5.29; found: C, 71.21; H, 5.21.

**4'-Cyano-[1,1'-biphenyl]-4-yl 3-((4-(octyloxy)-2-hydroxybenzylidene)amino)benzoate, (1d): **^1^H NMR (400 MHz, CDCl_3_) δ 13.40 (1H, s, -OH), 8.59 (1H, s, –CH=N–), 8.08 (2H, d, *J* = 7.2 Hz, ArH), 7.73 (2H, d, *J* = 8.1 Hz, ArH), 7.67 (2H, d, *J* = 9.0 Hz, ArH), 7.64 (2H, d, *J* = 8.7 Hz, ArH), 7.55 (1H, d, *J* = 7.6 Hz, ArH), 7.34 (2H, d, *J* = 8.4 Hz, ArH), 7.29 (2H,d, *J* = 8.7 Hz, ArH), 6.49 (2H, d, *J* = 2.4, 8.4 Hz, ArH), 3.99 (2H, t, *J* = 6.4 Hz, -O-CH_2_-), 1.81–1.28 (12H, m, -(CH_2_)_6_-), 0.87 (3H, t, -CH_3_); Anal. calcd for C_35_H_34_N_2_O_4_: C, 76.90; H, 6.27; found: C, 76.28; H, 6.15.

**4'-Cyano-[1,1'-biphenyl]-4-yl 3-((4-(hexyloxy)-2-hydroxybenzylidene)amino)-4-chlorobenzoate (1e):** IR (KBr) ν_max_: 3184, 2951, 2856, 2222, 1737, 1600, 1512, 1489, 1381, 1342, 1290, 1191, 1169, 1112, 746 cm^−1^; Anal. calcd for C_33_H_29_ClN_2_O_4_: C, 71.67; H, 5.29; found: C, 71.14; H, 5.24.

**4'-Cyanobiphenyl-4-yl 3-((4-(hexadecyloxy)-2-hydroxybenzylidene)amino)-4-chlorobenzoate (1f):** IR (KBr) ν_max_: 3184, 2951, 2856, 2222, 1737, 1600, 1488, 1389, 1290, 1215, 1192, 1170, 1112, 746 cm^−1^; Anal. calcd for C_43_H_49_ClN_2_O_4_: C, 74.49; H, 7.12; found: C, 74.18; H, 7.02.
